# Recent Progress in the Classification and Operation of Sacral Fractures

**DOI:** 10.1155/2023/2795722

**Published:** 2023-03-12

**Authors:** Nian Sun, Yijun Liu, Haohan Yan, Zhiqiang Zhang, Yanbing Li, Canjun Zeng

**Affiliations:** ^1^Department of Foot and Ankle Surgery, Center for Orthopaedic Surgery, The Third Affiliated Hospital of Southern Medical University, Guangzhou, China; ^2^Orthopaedic Hospital of Guangdong Province, Guangzhou, China; ^3^National Key Discipline of Human Anatomy, School of Basic Medical Sciences, Southern Medical University, Guangzhou 510000, China

## Abstract

Most sacral fractures are caused by high-energy, violent injuries, often accompanied by lumbosacral plexus injuries, which can cause instability of the posterior pelvic ring or lumbosacral junction in severe cases. Currently, the most commonly used clinical classification methods are Denis classification, Tile classification, Isler classification, and Denis II classification. In recent years, lumbosacral vertebral injury classification and injury degree scoring systems have often been applied clinically as the choice of treatment methods. At present, the internal fixation and implantation methods of sacral fracture are developing in the direction of positive, efficient, safe, and minimally invasive. But different fixation methods have their own indications, which should be strictly followed. This article reviews the classification of sacral fractures and the latest progress in surgical treatment.

## 1. Introduction

The sacrum is the center of the pelvic load, carrying the upper body load from the lumbar spine through the L5S1 intervertebral disc and the articular process above and transmitting the load through the sacroiliac joint to the lower limb or rami of the ischial bone. The sacrum needs to accommodate the changing load conduction from sitting to standing position. On the other hand, the sacrum is the most enlarged part of the spine. Together with the iliac crest, the sacrum forms the cornerstone of the whole spine, especially the lumbar movement, and is the axis of the upper body rotation during running and jumping. Therefore, the joint of the lumbar spine, sacrum, and iliac crest with the sacrum as the center requires both strong stability and elastic micromovement. It is the strongest and most complex ligamentous bone structure complex in the human body. Its irregular bone structure and interlacing dense ligament structure confuse people, and its biomechanical mechanism and characteristics are still not completely clear. Traumatic sacral fractures are mostly high-energy injuries, accounting for 1% of fractures and 20%∼30% of pelvic fractures. With the development of imaging technology, especially MRI, the reports of an exercise-induced stress fracture, senile osteoporotic fracture, and fracture after radiotherapy are increasing gradually. Treatment of sacral fractures includes both nonoperative and operative treatments. Nonsurgical fractures were based on rest, analgesic fractures, and early activity. Surgical treatment can be divided into three categories: percutaneous sacroiliac screw fixation, posterior pelvic fixation, and anterior pelvic internal fixation. As for the treatment of unstable sacral fractures, there are still differences on whether to choose surgical treatment as well as the method and timing of surgical treatment [1]. 95% of traumatic sacral fractures are associated with injuries, and common ones include sacral plexus injuries, fractures elsewhere in the pelvic ring, hip or lumbar fractures, pelvic organ injuries, sacrocaudal skin soft tissue injuries, and even open fractures (it is important to note that sacral fractures may be communicated through the damaged intestine). This article reviews and discusses the current status and latest progress of surgical treatment of sacral fractures.

## 2. Classification of Sacral Fractures

### 2.1. Fracture Types Involving Only the Sacrum

#### 2.1.1. Denis Parting

Denis et al. [2] divided the sacrum into three regions according to sacral anatomy. Region I: sacral wing region; Region II: sacral foramen region; Region III: sacral canal region. According to the anatomical location of the fracture, the fracture was classified into three types: ① Type I: The fracture line is located in the lateral region of the sacrum and is the most common type, accounting for about 50% of patients with this type of fracture. L5 nerve roots pass anteriorly and can be damaged by fractures. ② Type II: fracture line transsacral foramen; Zone II fractures are characterized by injuries to the L5 nerves through the lower sacral nerve heel. Patients with neurological symptoms account for 21% to 28%, and zone II fractures account for 34% to 47.5% of sacral fractures [3]. ③ Type III: fracture line through the sacral canal. The transverse fracture is type III because the fracture end passes through the sacral canal. Cauda equina syndrome involving sacral canal fracture displacement can damage the cauda equina, usually damaging bilateral nerve roots above S4, which is manifested as cauda equina syndrome, mainly involving intestinal dysfunction or bladder dysfunction [4]. The advantage of this classification is that it points out the risk of nerve damage in different fracture types but does not provide any guidance for surgical management.

#### 2.1.2. Roy—Camille Parting

Roy-Camille et al. [5] studied 13 patients with high sacral fractures and referred to the fractures above the S2 vertebral body as high sacral transverse fractures. Since most of the injuries were caused by falling from a high altitude, it was also called a “jump (suicide) fracture.” The lower sacral transverse fracture is the one below S2. For better clinical application, Roy-Camille et al. subdivided high sacral transverse fractures into the following three types according to the location of the lumbar spine at the time of injury: ① type I: angular without displacement; type II: angular and displaced; and ③ type III: The sacrum has complete displacement of the head and tail. In 1991, Strange-Vognsen and Lebech [6] reported a case of high sacral transverse fracture with obvious rectal and bladder symptoms. According to imaging analysis, it was a nondisplaced comminted fracture, and they added it to Roy–Camille type IV, that is, a neutral position fracture. The improved Roy–Camille classification method can be used to guide the classification of transverse fractures above S4, predict the risk of neurological damage, and provide guidance for surgical treatment.

#### 2.1.3. Gibbons Parting

Based on the Denis classification, Gibbons et al. [7] studied and analyzed 44 patients with sacral fractures in 1990. According to the degree of involvement of the sacral foramina and the sacral canal, the fracture line morphology was associated with a nerve injury, which was divided into a longitudinal fracture and a transverse fracture. This type is further subdivided into 4 types, but this type is basically similar to the Denis type, so it is not repeated here. It is important to note that longitudinal fractures often cause longitudinal instability of the pelvis, and transverse fractures often exhibit symptoms of severe nerve damage. In order to evaluate the recovery of nerve function before and after treatment, Gibbons et al. summarized a large number of cases and divided cauda equina injuries in sacral fractures into four types: ① type 1: no nerve damage; ② type 2: only paresthesia; ③ type 3: lower extremity movement impaired; and ④ type 4: intestinal or bladder dysfunction. The classification is of great significance for evaluating the degree of nerve injury and the recovery of the nerve before and after treatment.

### 2.2. Classification of Sacral Fractures in terms of Pelvic Fractures

#### 2.2.1. Tile Parting

In 1988, Tile [8] classified pelvic fractures into three categories according to the stability of pelvic fractures and the displacement direction of the fracture end, among which sacral fractures were involved in: ① type A (stable type): pelvic fractures that do not affect the stability of the pelvis and do not extend to the pelvic ring, such as low transverse fractures of the sacrum at S3 and below, isolated iliac wing fractures; ② type B2: lateral compression injury of the pelvis or internal rotation injury of the hip bone; the sacroiliac complex may be compressed due to a sacral fracture; ③ type B3 (barrel handle): the main fractures are located in the anterior ring with a contralateral posterior ring fracture, such as the left pubic ramus with an ischial ramus fracture and a posterior sacral compression fracture; ④ type C: a complex type of pelvic fracture with both rotational and vertical instability. Since vertical instability is involved, each subtype of type C can include a sacral fracture. This classification highlights the important role of the sacrum in the stability of the pelvic posterior ring and guides clinicians to view sacral fractures from the perspective of the overall stability of the pelvis.

#### 2.2.2. Young—Burgess

In 1990, Burgess et al. [9] further subdivided the Pennal classification based on the Pennal classification and summarized the Young-Burgess classification after analyzing 210 patients with pelvic fractures. The classification of different injury types is as follows: ① lateral extrusion (LC) can be divided into three types, among which the common general feature of LC (i–iii) is the transverse anterior fracture (upper and lower ramus of pubis). The distinguishing feature of LC I is a sacral compression fracture on the injured side. LC II was characterized by a half-moon fracture of the iliac wing. The distinguishing feature of LC III was a contralateral open-book injury. ② Anterior and posterior compression (APC) can also be divided into 3 types. The general feature of APC I is the separation of the pubic symphysis. The distinguishing features of APC I are: separation of the sacroiliac joint; the anterior and posterior ligaments of the sacroiliac joint are pulled but still intact; the common characteristic of APC (II-III) is the separation of the pubic symphysis or the longitudinal fracture of the anterior pelvic ring. The distinguishing features of APC II were the widening of the sacroiliac joint, the fracture of the anterior ligament, and the integrity of the posterior ligament. APC III was characterized by complete hemipelvic separation without longitudinal displacement, sacroiliac joint separation, iliac wing, or sacral fracture. ③ Vertical shear type (VS): the general characteristics are pubic symphysis separation or a longitudinal fracture of the pelvic anterior ring. The differential characteristics are longitudinal displacement and anteroposterior displacement of the pelvis, usually through the sacroiliac joint or the iliac crest. ④ Compound (CM), the general characteristics of the pelvic anterior ring, posterior ring longitudinal or transverse fracture, and the differential characteristics of longitudinal displacement and anteroposterior displacement, usually through the sacroiliac joint but also through the iliac crest mixed with other types. Burgess, think this kind of classification on the imaging model can also be passed on to the size and direction of iliac blood vessels and their branches around the force of the tag. The force will be around the sacroiliac joint ligament injury, causing internal organs and blood vessels to be damaged. According to the size and direction of the force, we can determine the weight of the patients with varying degrees of injury and, based on these special cases, offer treatment.

#### 2.2.3. AO

The AO classification of pelvic fractures formulated by AO/ASIF in its Fixation Manual in 1991 is basically the same as the Tile classification. The difference is that type B2 is divided into B2.1 lateral compression type (ipsilateral) and the B2.2 lateral compression type (barrel handle type). Type C1 is divided into C1.1 sacroiliac fracture, C1.2 sacroiliac dislocation or fracture dislocation, and C1.3 sacral fracture [10]. The AO classification is used to determine the stability of the pelvic ring in the presence of sacral fractures. Type A fractures with stable pelvic rings included coccygeal fractures and sacrococcygeal dislocation (AO/OTA: 61-A3.1); no displaced sacral fracture below S_2_ with no involvement of the pelvic ring (A0/OTA: 61-A3.2); and a displaced sacral fracture below S_2_ not involving the pelvic ring (A0/OTA: 61-A3.3). Type B fractures with unstable rotation of the pelvic ring include open sacrum fracture (AO/OTA: 61-B1.2AO/OTA: 61-CB3.1), unilateral lateral compression injury (AO/OTA: 61-B2.1), and bilateral lateral compression injury (AO/OTA: 61-B3.3). The vertical rotation of the pelvic ring was unstable for type C fractures, including unilateral sacral instability (AO/OTA: 61-C1.3); unilateral sacral fracture with contralateral posterior B-type pelvic ring injury (AO/OTA: 61-C2.3); and bilateral sacral fracture (height-fall injury) (AO/OTA: 61-C3.3).

### 2.3. Classification of Sacral Fractures from Lumbar-Pelvic Aspects

#### 2.3.1. Isler Parting

In 1990, Isler [11], through a retrospective analysis of 193 patients with pelvic fractures, found three different types of lumbosacral joint-related lesions, with the common feature that the fracture line traversed the S_1_ articular process. Type 1: fractures outside the L_5_/S_1_ facet: subluxation of the S_1_ facet or L_5_ subarticular process. Type 2: intraarticular L_5_/S_1_ fractures, such as sacral fractures through the S_1_ joint. Type 2 fractures are subdivided into type 3 fractures: ①2a: sacral fractures pass through the joints of S_1_, resulting in joint dislocation, but the degree of dislocation is small; ②2b: sacral fractures pass through the joints of S_1_, resulting in joint subluxation; ③2c: the sacral fracture passed within the S_1_ joint, resulting in complete dislocation. Type 3: this is a complex injury characterized by hemipelvic dislocation resulting in fractures of the articular process, interarticular, lamina, and pedicle, accompanied by dislocation of the L_5_ vertebra. This classification only analyzed the stability of the lumbosacral region but did not involve the stability of the pelvis or nerve damage. In order to be more applicable to clinical practice, Isler and Ganz [12] subdivided AO types A, B, and C into 3 categories and 3 subcategories based on the stability and integrity of the pelvic posterior ring in 1996 and added an anterior ring injury in addition to ABC type 3. In the classification of pelvic posterior ring injury and anterior ring injury, it is a complete classification of pelvic fracture showing the characteristics of pelvic ring injury. This classification is complicated and difficult to remember.

#### 2.3.2. Spine-Pelvis Separation

In 1996, Bents et al. [13] found an uncommon fracture type of sacrum in clinical work, which was different from stable lumbosacral junction fractures and dislocations in terms of severity and difficulty of patient management. Bents et al. defined a lumbosacral injury as a traumatic spine-pelvis separation involving a lumbosacral fracture with dislocation, a bilateral fracture or dislocation between the sacroiliac joints, and a sacral transverse fracture with bilateral sacroiliac joint dislocation. This type of injury is the combination of a transverse fracture and a longitudinal fracture, as defined by Gibbons et al. H and U fractures are common. Such injuries are usually caused by high-energy injuries and often lead to instability of the lumbar vertebrae and pelvis.

#### 2.3.3. Lumbosacral Injury Classification System

It was proposed by Lehman et al. [14] in 2012 to guide the surgical treatment of complex sacral fractures in the clinic. The lumbosacral injury classification system is a classification system designed after the spinal and lower cervical injury classification systems and the thoracolumbar injury classification system [15]. This classification is based on fracture morphology, the posterior sacroligament complex, and the presentation of the nerve injury. A summative score, called the cumulative injury severity score, is created from the three categories, with a total score between 1 and 10. If the total score is greater than 4, surgery is recommended. Equal to 4 points, according to the specific situation of the patient to decide; less than 4 points, conservative treatment is recommended. The details are as follows: fracture morphology: the kyphosis ≤20° was 1 min, and the kyphosis >20° was 2 min. Axial compression fractures: 2 without sacral foramen and canal involvement, 3 with sacral foramen and canal involvement, 3 with transverse displacement or rotational displacement, and 4 with burst or vertical fracture. The injuries to the posterior sacral composite ligament were 0 points complete, 1 point incomplete, and 2 points complete. Degree of nerve injury: no nerve injury (0 points); only paresthesia (1 point); lower limb motor dysfunction (2 points); intestinal or bladder dysfunction (3 points); progressive nerve injury (4 points); the total score 0–10 points. This classification system is novel but needs further validation before it can be widely used.

In addition, Schmidek et al. [16] and Sabiston and Wing [17] also classified sacral fractures. Among the many methods of sacral fracture classification, Denis and Tile are the most commonly used. However, each of the above classifications has its advantages in the diagnosis and treatment of fractures, but there is no lack of their one-sidedness. The importance of typing lies in guiding clinical treatment and providing a theoretical basis for the next treatment. As a clinical worker to flexibly use the knowledge, the patient's actual condition, and theoretical knowledge in a comprehensive analysis in order to achieve the best curative effect.

## 3. Surgical Treatment of Sacral Fractures

### 3.1. Percutaneous Sacroiliac Screw Fixation

The best indications for sacroiliac screw fixation are sacroiliac dislocation and Denis zone 1 fracture. However, the use of comminuted Denis zones 2 and 3 sacral fractures may be limited, and proximal anatomical reduction of the fracture is required before screw placement. Many scholars have studied the anatomical application of SIJS and how to place them safely. Coskun [18] et al. divided the medial and lateral sacral safety zones, and the average medial safety zone measured on MRI of 400 patients was 32.8 mm. The average lateral safety zone was 17.7 mm. Although sacroiliac screw fixation is a well-established technique, there are anatomical variations in the sacrum, and vascular and nerve injuries have been reported frequently during screw placement. In addition, due to the small space available for S_2_ vertebral placement, the risk of nerve injury is much higher than that of S_1_ vertebral placement [19]. To solve this problem, Bagheri H. et al. [20] measured the S_1_-S_2_ vertebrae, pedicle, sacral foramen, and sacral canal of 87 sacrum bones. The pedicle depth of S_1_ was 25.8 ± 2.3 mm, and the sacral alar depth was 50.1 ± 1.7 mm. The measured anteromedial angle of the pedicle was 29.6° ± 0.9°, and the sacral alar angle was 29.7° ± 2.1°. This is helpful for the clinical placement of sacroiliac screws.

In addition, the comparison of the effectiveness and safety of SIJS and other internal fixation methods for sacral fractures is a research hotspot. Wenning et al. [21] reported the differences in intraoperative operation, complications, and postoperative range of motion between sacroiliac screws and spinopelvic fixation. Twenty-nine patients and 48 patients received sacroiliac screws and spinopelvic fixation, respectively. The spinopelvic fixation group had earlier weight-bearing, but the sacroiliac screw group had shorter operation times, shorter hospital stays, and lower postoperative wound infection rates. Computer 3D navigation and robotics make the application of SIJS more convenient. It has been reported [22] that the use of 3D fluoroscopic navigation (see the procedure chart) has a lower iliosacral screw misalignment rate than conventional techniques or 2D fluoroscopic navigation. In addition, it reduces radiation exposure and the correction rate compared to conventional techniques. However, the misalignment rate associated with 3D perspective navigation is between 0% and 31%, indicating that there is still room for improvement in navigation performance. With the application of CT-aided design and computer three-dimensional navigation technology, nail placement is more accurate, intraoperative fluoroscopy times are less, the operation time is shorter, and the incidence of postoperative complications is lower. Weil et al. [23] used A Renaissance robot mounted on A multidirectional bridge connected to the patient's spine and then planned the implant trajectory during preoperative or intraoperative 3D scans. Placing screw after inserting guide wire percutaneously. The accuracy of sacroiliac screw placement, operation time, and fluoroscopy time were evaluated, and it was pointed out that the robotic system can implant internal fixators accurately and accurately for sacral fractures, which is a safe and reproducible method. Wu et al. [24] designed a new 3D template. The new guidance template was attached to the iliac crest on the pelvic model before operation and then installed on the internal and external cannulas on the base to insert the Kirschner wire into the model. Sacroiliac screw placement was assisted by a 3D-printed template in 19 patients, and IS screws were directly inserted under fluoroscopy in 18 patients. The investigators compared the quality of reduction, screw grade, operation time, and number of radiation exposures between the two groups and found that the combined template-assisted sacroiliac screw placement had higher accuracy, less fluoroscopy, shorter operation time, and avoided neurovascular injury caused by screw dislocation. Balling [25], under the guidance of 3D images, applied 120 navigation screws to 124 sacral fracture sites (the first and second vertebrae) of 52 patients. After 2 years of follow-up, the average visual analogue scale (VAS) decreased from 8.9 ± 1.1 preoperatively (3.6 ± 1.7 postoperatively) to 1.8 ± 1.9. The OSwestry Disability index (ODI) increased from 86.2 ± 4.9% preoperatively (28.5 ± 9.5% postoperatively) to 23.3 ± 13.7%. Krappinger et al. [26] proposed the use of preoperative CT scanning to develop a starting point and safe trajectory plan for safe and accurate fluoroscopic control of percutaneous iliosacral screw placement (SI screw) and used this technique to place 59 screws in 34 patients. Satisfactory outcomes were achieved in 97% of the patients. Richter et al. [27] used a fixed robotic 3D plate detector and a navigation system connected to the operating table for intraoperative navigation to improve the precision of SI screws. Fernandez-Fernandez et al. [28] 11 patients with complex posterior ring fracture combined with vertical instability were treated with iliac screw under CT localization. The mean follow-up time was 33 months. Among them, 9 cases had an excellent Matta score, and 2 cases had a good Matta score. The asymmetry index increased from 13.18 to 2.72. The deformity index increased from 0.049 to 0.010. Seven patients were able to resume their previous activities. Only 2 patients with Denis 2 fractures had secondary displacement during follow-up. Four patients developed neurological complications as a result of the initial injury. Percutaneous transsacral screw fixation has become a popular fixation method due to its low complication rate and good clinical results. It has been suggested that partially threaded nails may cause iatrogenic nerve damage. Herman et al. [29] performed a retrospective study on 90 patients who received partially threaded screws under the assumption that they would not cause iatrogenic nerve damage, of which only 4 patients still had neurological dysfunction at the last follow-up. With the in-depth study of sacral anatomy, the progress of SJUS placement technology, and the development of artificial intelligence, percutaneous sacroiliac screw fixation (SIJS), which uses a minimally invasive method to fix screws to the S_1_ or S_2_ sacral vertebrae via the iliac bone, has been widely accepted due to the advantages of less bleeding and low complications.

Currently, the common clinical treatment methods for Denis II sacral fracture and pelvic anterior ring fracture include open reduction plate fixation of the pelvic anterior ring fracture, ilio-lumbar pedicle screw internal fixation combined with external fixation, ilio-lumbar pedicle screw combined with pelvic anterior ring plate internal fixation, etc. Anterior pelvic ring plate internal fixation is only the reduction and fixation of anterior pelvic ring fracture. Through reliable reduction and fixation of the anterior ring fracture, indirect reduction of the posterior pelvic ring can achieve the purpose of sacral nerve decompression to a certain extent. Although open reduction of the anterior pelvic ring has relatively small trauma, its stability is poor, and there is no reliable three-dimensional fixation of the lumbosacral part and the entire pelvic ring, nor can it completely decompress the injured sacral nerve. Although pedicle screw internal fixation combined with external fixation can effectively fix the anterior and posterior pelvic rings, the external fixation is difficult to care for, and the risk of infection is high, so it is difficult to obtain a satisfactory effect for the reduction of the anterior ring comminted fracture. It has been reported that external fixators can temporarily fix pelvic fractures, but using external fixators as the final fixation method can lead to severe malunion, with a malunion rate of up to 50%. Ilio-lumbar pedicle screw combined with anterior pelvic ring plate internal fixation is satisfactory for fracture reduction, can better solve the disadvantages of simple use of anterior ring plate fixation or ilio-lumbar pedicle screw internal fixation, and has the following obvious advantages: (1) The posterior approach to surgery has clear visual field, large operating space, solid internal fixation, large orthopedic force, good reduction effect on the sacrum, can restore the normal anatomical structure of the sacroiliac, and good indirect reduction effect on the anterior pelvic ring. (2) The posterior internal fixation reduction can clearly explore the sacral nerve injury, fully relieve the sacral nerve compression, and provide a safe space for the sacral nerve. (3) It can not only restore the closed ring structure of the pelvis but also restore normal spine-pelvis physiological force line conduction. (4) It is a three-dimensional fixation of the spine and pelvis, which can limit fracture displacement and avoid further injury of sacral nerve. (5) In line with the anatomical and physiological structure of the spine and pelvis, fixation is effective and reliable, so patients can get out of bed early and reduce bed complications. The results of the study by Zhang et al. [30] showed that all the patients in the study group had solid fracture fixation, no displacement, and no loosening or fracture of the nail rod and plate during the follow-up.

### 3.2. Posterior Fixation Technique

#### 3.2.1. Spinal Pelvic Internal Fixation

Throughout the history of posterior spinal pelopexy, postoperative wound exudation, incision infection, and foreign body sensation caused by internal fixators have always been difficult problems for surgeons. Traditional open spinopelvic fixation has drawbacks and complications, such as relatively high infection rates (10 to 15%), wound dehiscence, and instrumentation problems. Complications associated with wounds are relatively common due to overexposure. Two reports have shown the advantages of minimally invasive spinopelvic fixation over traditional incisions. With the development of artificial technology, minimally invasive internal fixation combined with computer and robot navigation has been increasingly applied in the treatment of orthopedic patients. Liu et al. [31] treated 12 patients with traumatic spinopelvic separation in a single center since March 2016, all of whom were treated with minimally invasive lumbar and pelvic fixation assisted by the third-generation Tianji orthopedic robot (TINAVI Medical Technologies, Beijing, China). Compared with the control group, surgery time is shorter, intraoperative bleeding is less, average length of hospital stay is shorter, and complication rates are lower. Tsai et al. [32] reported the imaging and clinical results of a single-center retrospective study with more than 1 year of follow-up, in which 21 patients and 17 patients were treated with traditional open fixation and minimally invasive fixation, respectively. There were no significant differences in postoperative imaging assessment and functional scores between the two groups. The minimally invasive fixation group had shorter operation time, less intraoperative bleeding, and a lower incidence of complications. Compared with the traditional open surgery group, the postoperative appearance is more beautiful. Decker et al. [33] also proposed a minimally invasive U-shaped spinopelvic stabilization technique: LPS from L3/4 to the iliac crest with a robust cross-rod/cross-connector between the iliac crest screws. Two iliac crest screws were connected with a 5.5 mm transverse rod. A 5.5 mm transverse connector was used to attach to the pedicle screw. There were no soft tissue-related complications in the 10 patients who underwent the operation, and there was no abnormal injury in postoperative function and mobility. Koshimune et al. [34] reported the results of minimally invasive spinopelvic fixation for the treatment of unstable bilateral sacral fractures. In this study, three of the eight patients who received conventional fixation had MRSA infections, while those who received minimally invasive fixation did not. Of the 16 patients, 15 had bone union. In conclusion, minimally invasive spinopelopexy is associated with less operative time, less bleeding, and lower infection rates than traditional open spinopelopexy. Many scholars have compared spinopelvic fixation with other fixation techniques. Shetty et al. [35] reported the imaging and clinical results of a single-center retrospective study followed up for more than 2 years, in which 40 cases of noncomminuted longitudinal fractures with normal neurological function and satisfactory closed reduction were treated with sacroiliac screw internal fixation. Twenty-seven cases of comminuted or high transverse fractures with malformed anatomy or nerve loss were treated with spinopelvic fixation. It is pointed out that spinopelvic fixation techniques can be used for comminuted fractures, including vertical instability, unacceptable closed reduction, neurological dysfunction, lumbar deformity, and high transverse fractures. Spinopelvic fixation also has its disadvantages. Petryla et al. [36] followed up 16 patients with pelvic and spinal separation in a single center for 1 year and pointed out that only one-third of patients with pelvic and spinal separation reached the preinjury level one year after injury. Spinopelopexy has been continuously improved since it was shown to provide good reduction and sufficient strength for bilateral sacral fractures with vertical instability. Prost et al. [37] proposed minimally invasive lumbar iliac and iliosacral fixation techniques to treat posterior pelvic ring injuries. The operation was performed with the patient lying prone. This included the insertion of pedicle screws into L4 or L5 and fluoroscopy-guided screw fixation of the iliac crest. Intraoperative distraction can be performed according to the amount of displacement. An iliosacral screw was then inserted percutaneously to reset it in the transverse plane, creating a triangular structure. Okuda et al. [38] proposed crab-shaped internal fixation: percutaneous pedicle screws were bilaterally inserted into the L5 or L4 pedicle; 4 iliac screws were bilaterally inserted into the iliac crest; and titanium rods were used to connect the screw directions from the left to the right and from the head to the tail. This provides a feasible method for pelvic and spinal internal fixation, which can reduce the vertical displacement and bone healing of unstable pelvic ring fractures. Peng et al. [39] combined the Starr Frame navigation robot and the Da Vinci robot for minimally invasive surgical treatment of pelvic fractures. This treatment can be used to treat pelvic fractures and sacral nerve injuries, as well as early and late nerve repair for sacral fractures.

#### 3.2.2. Triangle Fixation Technique

The posterior pelvic ring fracture is treated with a spinal and pelvic longitudinal fixation system combined with a transverse sacroiliac screw or plate, which is called the “triangle fixation technique.” This technique has been proven to be the most biomechanically stable, providing multiplanar mechanical stability and enabling patients to walk with early loading. The disadvantage lies in the need for extensive dissection of paravertebral muscles, trauma, bleeding, postoperative wound exudate infection, and other problems. Jindal et al. [40] reported the early and midterm efficacy of spinal pelvic internal fixation and transverse iliac screw internal fixation in the treatment of unstable transsacral fractures. This investigator reported the radiological and clinical findings of a single-center retrospective study with a mean follow-up of 3.1 years in which 22 patients underwent trigonometry and noted that trigonometry is also a reliable treatment for unstable transsacral fractures that allows for early weight-bearing and promote faster functional recovery. Tian et al. [41] described a modified TOS procedure for the reduction and fixation of unstable vertical sacral fractures based on the principle of TOS. It consists of vertical and transverse rods and a pedicle screw system for fixation. In this report, the clinical outcomes were investigated radiologically, and the reduction and fixation of vertically unstable sacral fractures were examined to further assess the biomechanical function and surgery-related complications after final reduction and stabilization. Kanezaki et al. [42] proposed a minimally invasive trigonometry and followed up on the clinical outcomes of 10 patients with unstable sacral fractures treated with minimally invasive trigonometry (MITO) for at least 1 year. According to the Majeed functional rating system, the clinical outcomes of 8 patients were “excellent.” Tian et al. [43] 18 patients with traumatic spine-pelvis separation were treated with modified bilateral triangular fixation. After an average follow-up of 11 months, the excellent and good rate of Matta function was 89%. Therefore, Tian et al. believed that modified triangular internal fixation combined with internal fixation is an effective and advanced surgical option for patients with traumatic spine-pelvis separation.

#### 3.2.3. Other Fixation Methods

In addition to spinopelvic fixation and triangular fixation, Joo and Grauer [44] pointed out that the use of posterior superior margin screws as an auxiliary fixation method is a feasible choice and can be used as a reference for fixation scaffolds in complex pelvic and spinal reconstruction. A technical report describing posterior pedicle plates was provided by Boudissa et al. [45], which evaluated the clinical and radiological outcomes of 10 patients with vertically unstable pelvic fractures. The excellent and good rate of fracture reduction was 70%, the excellent and good rate of the Hannover and Majeed scores was 80%, and the average Majeed score was 71.8 ± 17 points. Complications included early postoperative sepsis requiring surgical irrigation in 3 cases and stent removal in 4 cases due to discomfort. Through biomechanical tests, Shinohara et al. [46] believed that the posterior internal fixation of the spine had more biomechanical strength than the traditional posterior plate fixation. For the treatment of longitudinal sacral fractures, Fathy Saoud et al. [47] proposed that percutaneous iliac screw fixation with the posterior superior iliac spine as the starting point is a new option for the treatment of sacral fractures. Fifty patients with longitudinal sacral fractures received “ileal” internal fixation. The investigators believe that minimally invasive “ileal” fixation provides a safe, rapid, and easy fixation method for such fractures. It is suitable for critically ill patients because it is fast and causes less blood loss. Tile C pelvic ring injuries are challenging for surgeons to manage. Most of these injuries can be managed by percutaneous reduction techniques, and the posterior ring can be stabilized by percutaneous transpedicle screw fixation. However, for a significant Denis II comminuted area or significant lateral/vertical displacement of the semipelvis through a complete sacral fracture. In this case, percutaneous treatment can be dangerous. Martin et al. [48] proposed a minimally invasive technique for indirect reduction and temporary stabilization that is soft tissue-friendly and allows the state of reduction to be maintained during final fixation surgery.

### 3.3. Front Fixation Technology

The anterior approach was considered a forbidden area for the surgical treatment of sacral fractures in the past because of its complex anatomy, difficult exposure, small operating space, and risk of injury to the lumbosacral trunk, sacral plexus, and blood vessels. Previously, scholars at home and abroad also performed presacral anatomy: the area of 2 cm saved from the lateral part of the L4 nerve to the sacroiliac pass was a safe area for surgery, and plate and screw fixation could be performed. Therefore, anterior plate fixation was initially indicated only for sacroiliac dislocations and partial Denis I fractures. In recent years, Huang et al. reported [49] that the modified para-rectus abdominis approach was used to fix the anterior sacral plate, and the common iliac arteries and veins and the lumbosacral plexus were safely raised and retracted with a rubber catheter to protect the common iliac arteries and veins and the lumbosacral plexus under direct sight during the operation, forming the “fifth window” of the modified para-rectus abdominis approach. The two ends of the pelvic reduction forceps can be placed on sacral cone 1 and the iliac crest to reduce the sacral fracture. The two plates are fixed simultaneously on the first cone, the sacral wing, and across the sacroiliac joint to the iliac crest to complete the sacral fracture fixation. This technique innovatively fixes the plate to the sacral 1 cone, broadens the scope and indications of anterior plate fixation for sacral fractures, and provides a new fixation idea for the anterior treatment of traumatic sacral fractures. Management of LC-1 pelvic injuries, especially in patients with a complete sacral fracture (LC-1PICSF, OTA 61-B2.1), remains controversial. The specific indications for fixation alone are unknown, and outcome data are scarce compared with combined fixation. Huang et al.[49] reported the imaging and clinical results of a single-center retrospective study with more than 2 years of follow-up. Thirty-six patients and 32 patients were treated with anterior internal fixation alone or combined anterior and posterior fixation, respectively. There were no significant differences between the two groups. Compared with combined fixation treatment, anterior fixation alone has a shorter operation time, less intraoperative fluoroscopy, and less intraoperative bleeding. Compared with anterior fixation alone, patients treated with combined fixation achieved full weight-bearing earlier.

## 4. Summary and Prospect

At present, the surgical treatment of unstable sacral fractures is a major challenge. The timing of surgery and fixation methods are still controversial, but the overall trend is towards minimally invasive treatment. The authors concluded that as a new anterior sacral approach for the treatment of sacral fractures, the para-rectus abdominis approach has the advantages of a small skin incision, satisfactory exposure, no need to cut muscle, short wound closure time, no nerve traction injury, less bleeding, and a low infection rate. Exploration and decompression of the superior sacral plexus (L4 to S1) in the presacral region have a significant visual field advantage, which can remove scar tissue under direct vision and reduce or remove free bone debris. It is a feasible treatment method without further destabilizing the posterior pelvic ring. However, for the inferior sacral plexus (S2 to S4), sacral nerve injury, and lumbosacral separation, a combined anterior and posterior approach is recommended for nerve exploration and decompression and fracture fixation due to the complex anatomical structure of the anterior sacral venous plexus, the difficulty of anterior sacral nerve exploration and decompression, and the inability to effectively stabilize the lumbosacral joint.

## Data Availability

The data used to support the findings of this study are included within the article.
